# Supplementary material for: Oncogenic G12D mutation alters local conformations and dynamics of K-Ras
Source: Sci Rep. 2019 Aug 13;9:11730. doi: 10.1038/s41598-019-48029-z (PMC6692342; doi:10.1038/s41598-019-48029-z)
Supplement: Supplementary file 1 — Supplementary Information [file 41598_2019_48029_MOESM1_ESM.pdf]

SUPPLEMENTARY

Oncogenic G12D mutation alters local conformations and dynamics of K-Ras

Sezen Vatansever[1,2], Burak Erman[3]*, Zeynep H. Gümüş[1, 2]*

[1]Department of Genetics and Genomic Sciences, Icahn School of Medicine at Mount Sinai, New York, New York, United States of America

[2] Icahn Institute for Data Science and Genomic Technology, New York, New York, United States of America

[3]Department of Chemical and Biological Engineering, College of Engineering, Koç University, Istanbul, Turkey

* Corresponding authors

E-mail: berman@ku.edu.tr (BE) and zeynep.gumus@mssm.edu (ZHG)

Supplementary Methods

MD Simulations

We solvated each protein in a TIP3P water box with 12Å buffering distance. We applied periodic boundary conditions and added ions to neutralize the system. We used a 2fs time-step with a 12Å cutoff for Van der Waals interactions and full particle-mesh Ewald electrostatics with 1Å grid spacing and direct space tolerance of 10^{-6}. We carried out all computations in N, P, T dynamics procedure. System temperature was kept constant at the physiological value of 310K using Langevin dynamics with a damping coefficient of $2ps^{-1}$. Constant pressure of 1atm was maintained by The Nose-Hoover Langevin piston method with a 200fs piston period and 100fs decay time.

Distance distributions between residue pairs

We calculated pairwise distances R_{ij} and their distributions $W(R_{ij})$ using the same methods in our previous paper[1] where R_{ij} formula is $\sqrt{(R_i(t) - R_j(t))^2}$ and the normalized $W(R_{ij})$ graphs were created by dividing the maximum R_{ij} value into small bins and counting the number of observed distances in each bin.

Calculation of dispersion of distributions of residue-pair distances

We calculated the probability function of distributions of residue pairs in the Figures 3-5, S1. For this purpose we used Matlab pdf() function which fits distributions to residue-pair distances. We recorded the mean values of the normal distributions (mu) and the standard deviation values of the normal distributions (∑) into the Table S2.

Pairwise correlation calculations

Correlations between the residue fluctuations $C(\Delta R_i, \Delta R_j)$ are calculated by the formula below[1]:

$$C_{ij} = C(\mathit{\Delta} Ri, \mathit{\Delta} Rj) = \frac{\langle \Delta R_i(t) \cdot \Delta R_j(t) \rangle}{\langle (\Delta R_i(t))^2 \rangle^{1/2} \langle (\Delta R_j(t))^2 \rangle^{1/2}} = \frac{\sum_{t=1}^{N_t-\tau} \Delta R_i(t) \cdot \Delta R_j(t)}{\left[\sum_{t=1}^{N_t-\tau} (\Delta {R_i}^2\,(t))\right]^{1/2} \left[\sum_{t=1}^{N_t-\tau} (\Delta {R_j}^2\,(t))\right]^{1/2}}$$

The sign · denotes the dot product.

Supplementary Figures

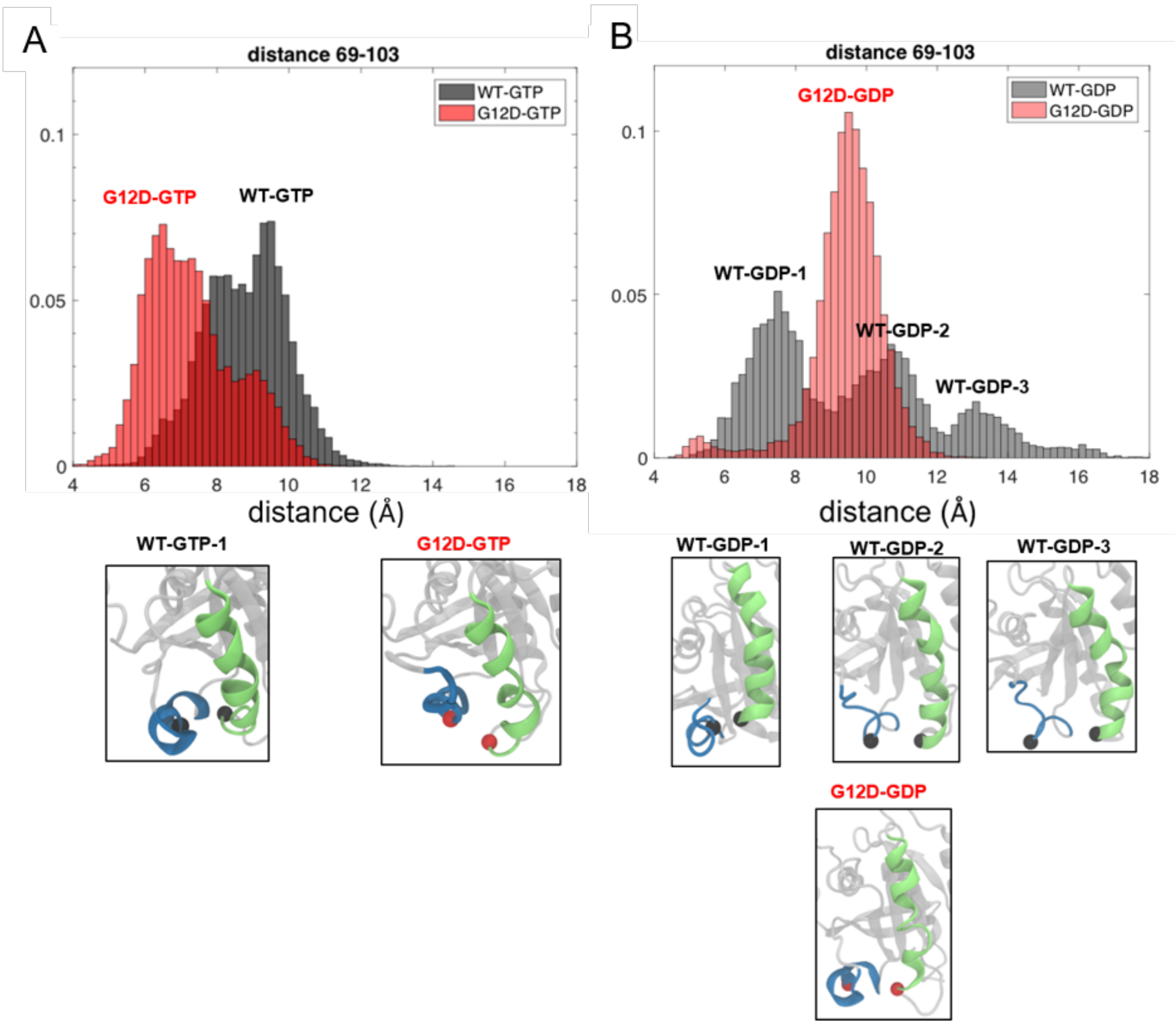

Figure S1. Distance distributions of the residue pair D69-V103 and the representative structures of SII (blue) and α3 (green) (A) K-Ras^{WT}-GTP (black) and K-Ras^{G12D}-GTP (red) (B) K-Ras^{WT}-GDP (grey) and K-Ras^{G12D}-GDP.

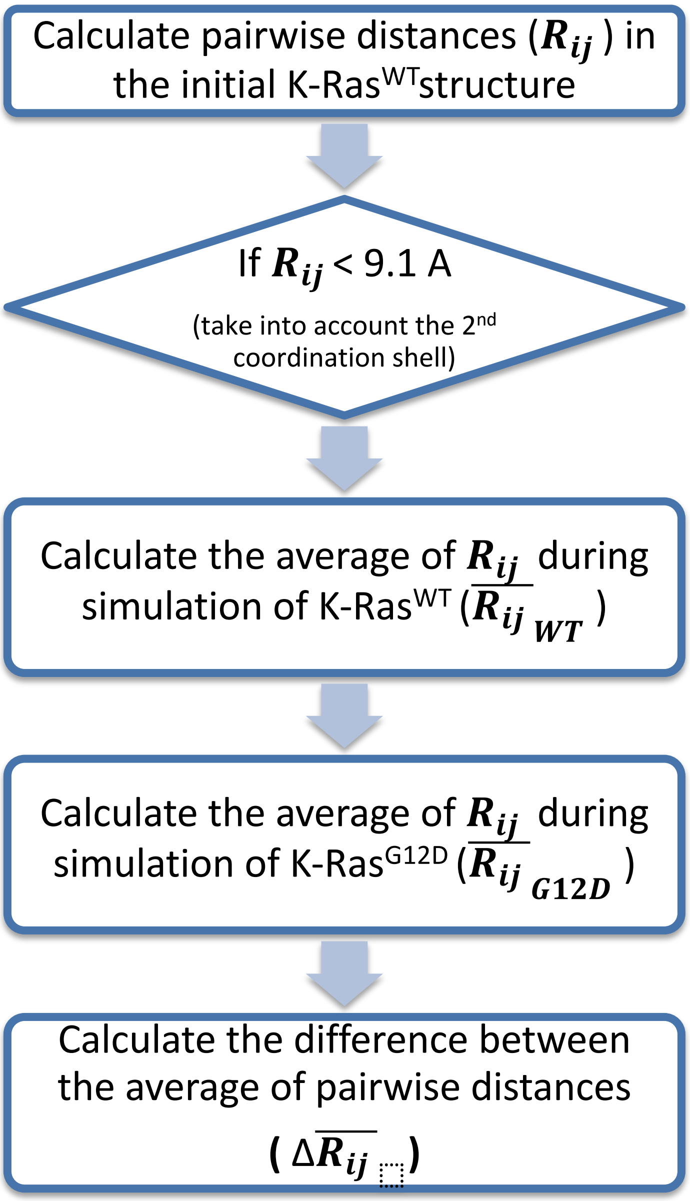

Figure S2. The algorithmic framework for calculating the change of distances between residue pairs ($\Delta\overline{R}_{ij}$) upon G12D mutation.

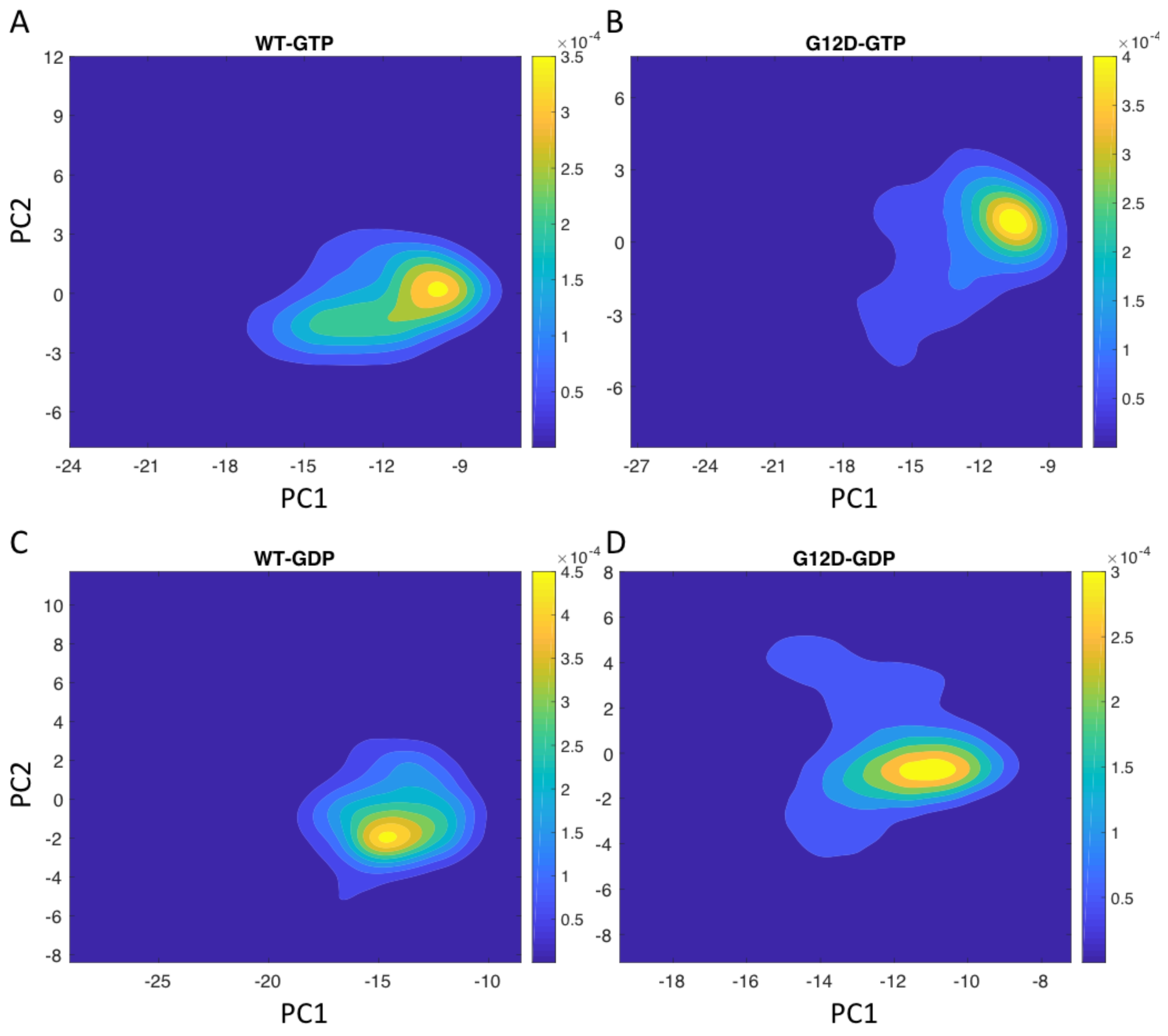

Figure S3. Projection of MD trajectories onto principal components. X axis is the first mode (PC1) and Y axis is the second mode (PC2). A) K-Ras^{WT}-GTP B) K-Ras^{G12D}-GTP C) K-Ras^{WT}-GDP D) K-Ras^{G12D}-GDP.

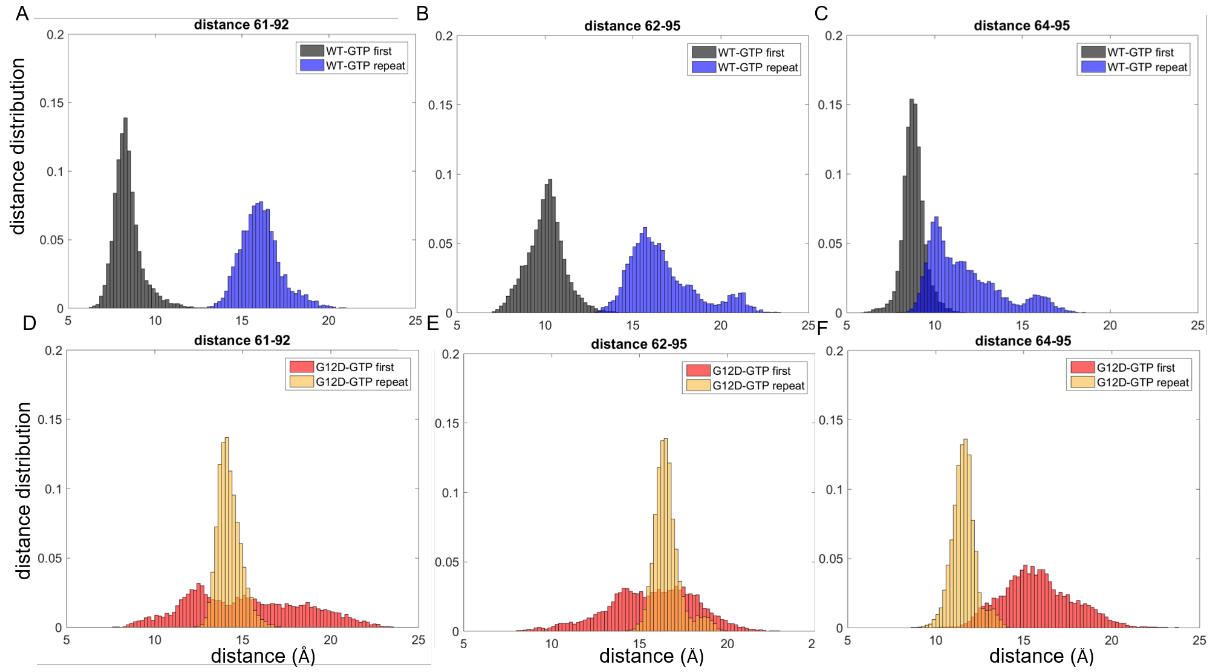

Figure S4 (Related to Figure 3A-C in the paper). Distribution of distances between residue pairs in SII-α3 region in each simulation of K-Ras-GTP. Distance distributions of Cα of residue pairs in K-Ras^{WT}-GTP first simulation (black) and K-Ras^{WT}-GTP repeat simulation (blue) (A) Q61-D92, (B) E62-H95, (C) Y64-H95; in K-Ras^{G12D}-GTP first simulation (red) and K-Ras^{G12D}-GTP repeat simulation (orange) (D) Q61-D92, (E) E62-H95, (F) Y64-H95.

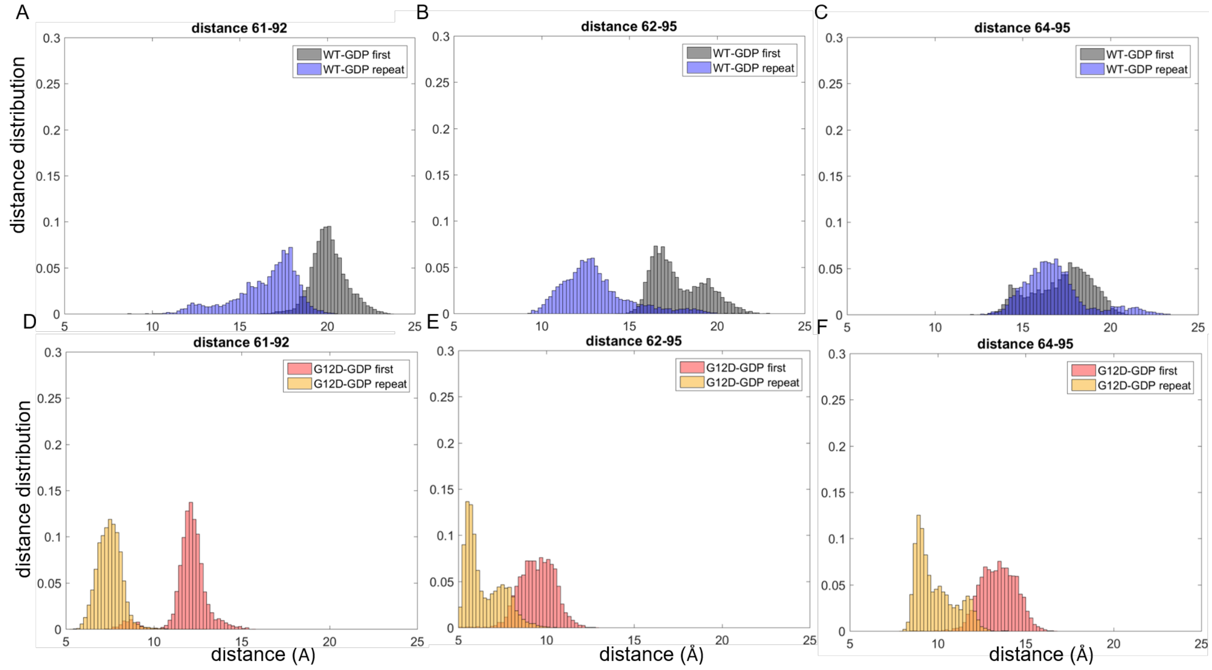

Figure S5 (Related to Figure 3D-F in the paper). Distribution of distances between residue pairs in SII-α3 region in each simulation of K-Ras-GDP. Distance distributions of Cα of residue pairs in K-Ras^{WT}-GDP first simulation (gray) and K-Ras^{WT}-GDP repeat simulation (light blue) (A) Q61-D92, (B) E62-H95, (C) Y64-H95; in K-Ras^{G12D}-GDP first simulation (pink) and K-Ras^{G12D}-GDP repeat simulation (yellow) (D) Q61-D92, (E) E62-H95, (F) Y64-H95.

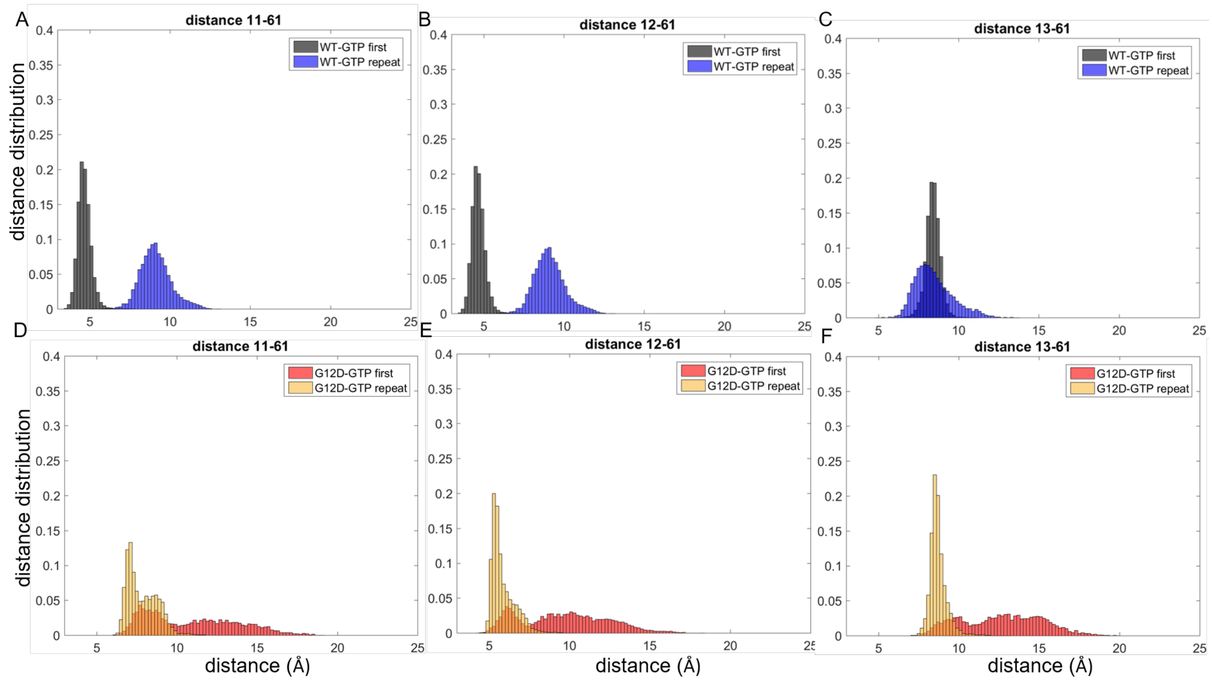

Figure S6 (Related to Figure 4A-C in the paper). Distance distributions between residue pairs in the P loop-SII region in each simulation of K-Ras-GTP. Distance distributions of Cα of residue pairs in K-Ras^{WT}-GTP first simulation (black) and K-Ras^{WT}-GTP repeat simulation (blue) (A) A11-Q61, (B) G12D-Q61, (C) G12D-Q61; in K-Ras^{G12D}-GTP first simulation (red) and K-Ras^{G12D}-GTP repeat simulation (orange) (D) A11-Q61, (E) G12D-Q61, (F) G12D-Q61.

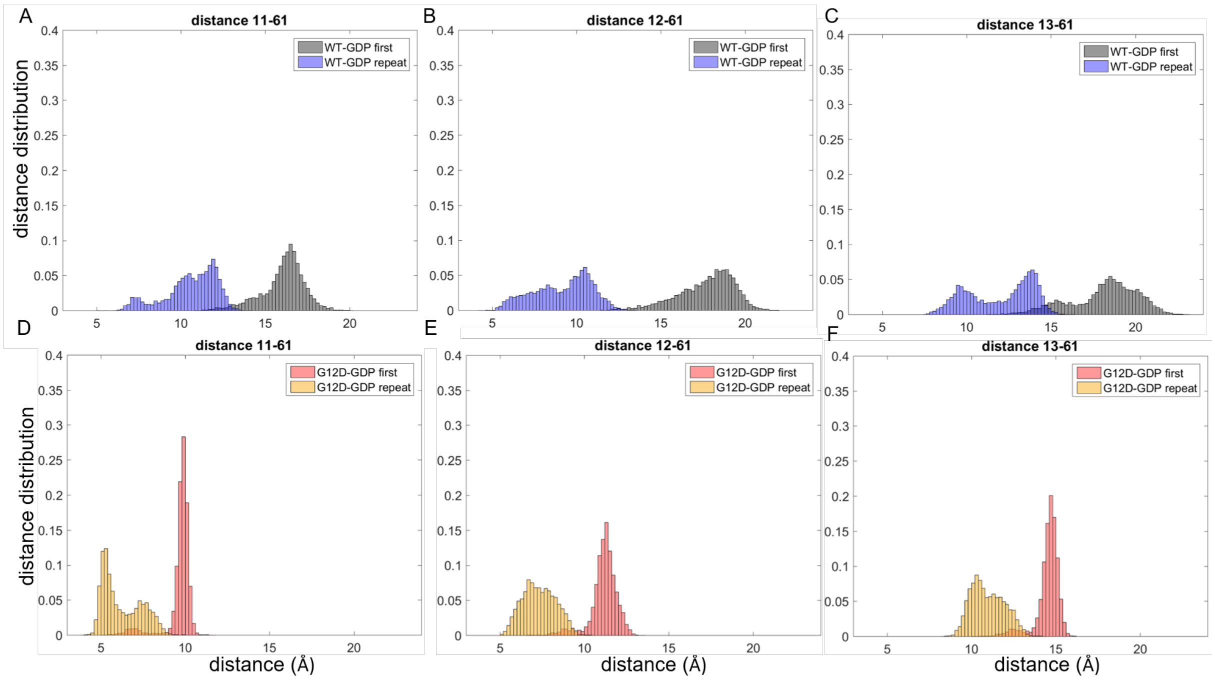

Figure S7 (Related to Figure 4D-F in the paper). Distance distributions between residue pairs in P loop-SII region in each simulation of K-Ras-GDP. Distance distributions of Cα of residue pairs in K-Ras^{WT}-GDP first simulation (gray) and K-Ras^{WT}-GDP repeat simulation (light blue) (A) (A) A11-Q61, (B) G12D-Q61, (C) G12D-Q61; in K-Ras^{G12D}-GDP first simulation (pink) and K-Ras^{G12D}-GDP repeat simulation (yellow) (D) A11-Q61, (E) G12D-Q61, (F) G12D-Q61.

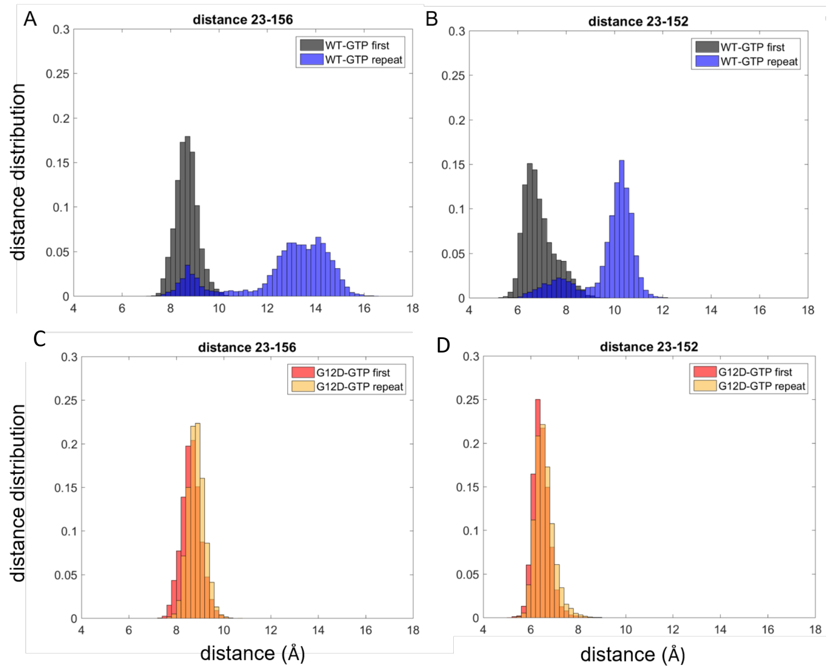

Figure S8 (Related to Figure 5A-B in the paper). Distance distributions of residue pairs which get closer after G12D mutation in each simulation of K-Ras-GTP. Distance distributions of Cα of residue pairs in K-Ras^{WT}-GTP first simulation (black) and K-Ras^{WT}-GTP repeat simulation (blue) (A) L23-V152, (B) L23-F156; in K-Ras^{G12D}-GTP first simulation (red) and K-Ras^{G12D}-GTP repeat simulation (orange) (C) L23-V152.

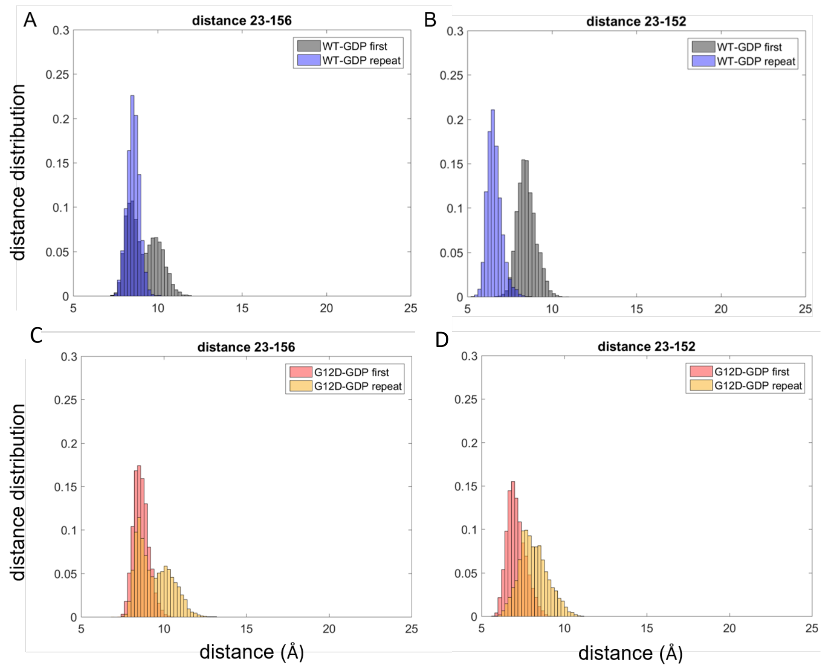

Figure S9 (Related to Figure 5C-D in the paper). Distance distributions of residue pairs which get closer after G12D mutation in each simulation of K-Ras-GDP. Distance distributions of Cα of residue pairs in K-Ras^{WT}-GDP first simulation (gray) and K-Ras^{WT}-GDP repeat simulation (light blue) (A) L23-V152, (B) L23-F156; in K-Ras^{G12D}-GDP first simulation (pink) and K-Ras^{G12D}-GDP repeat simulation (yellow) (C) L23-V152, (D) L23-F156.

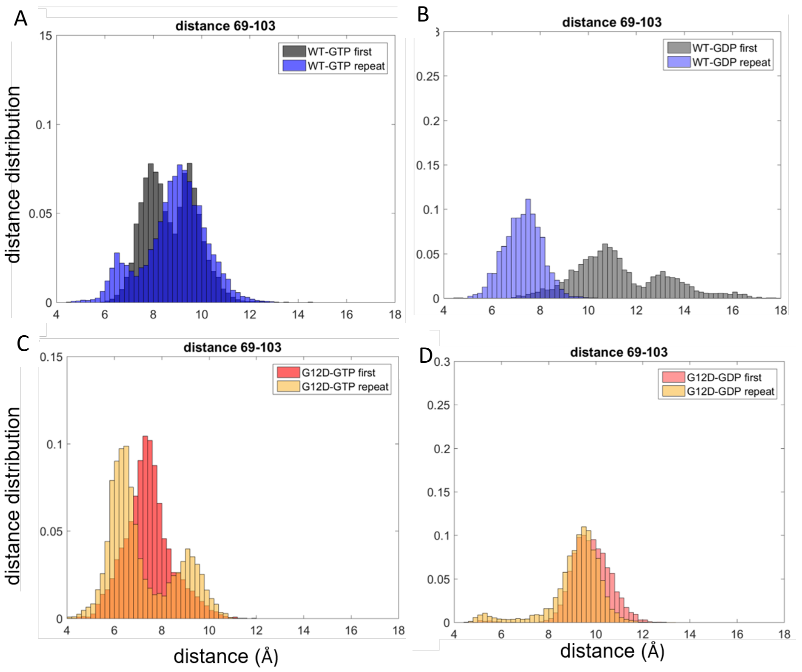

Figure S10 (Related to Figure S1). Distance distributions of the residue pairs D69-V103 in each simulation of K-Ras-GTP and K-Ras-GDP. Distance distributions of Cα of residue pairs in (A) K-Ras^{WT}-GTP first simulation (black) and K-Ras^{WT}-GTP repeat simulation (blue), (B) K-Ras^{WT}-GDP first simulation (gray) and K-Ras^{WT}-GDP repeat simulation (light blue), (C) K-Ras^{G12D}-GTP first simulation (red) and K-Ras^{G12D}-GTP repeat simulation (orange), (D) K-Ras^{G12D}-GDP first simulation (pink) and K-Ras^{G12D}-GDP repeat simulation (yellow).

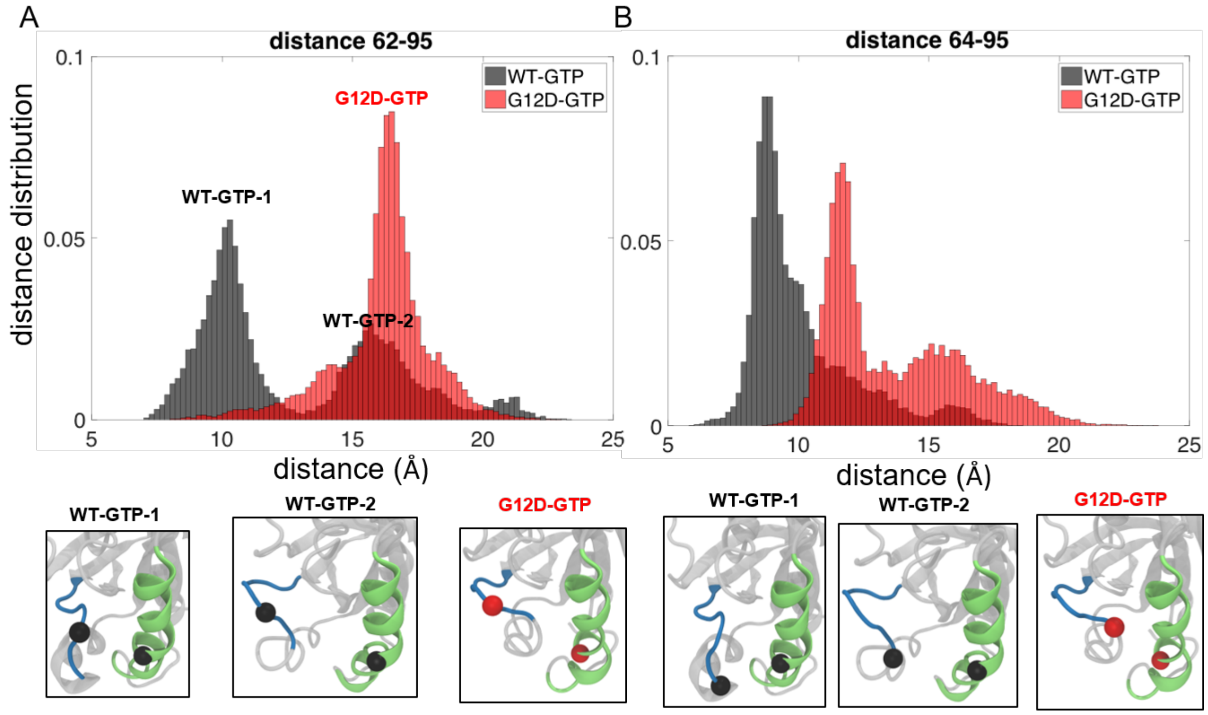

Figure S11 (Related to Figure 3B-C in the paper). Distance distributions between residue pairs in SII- α3 region in K-Ras^{WT}-GTP (black) and K-Ras^{G12D}-GTP (red) and representative structures of SII (blue)-α3 (green) (A) E62-H95, (B) Y64-H95.

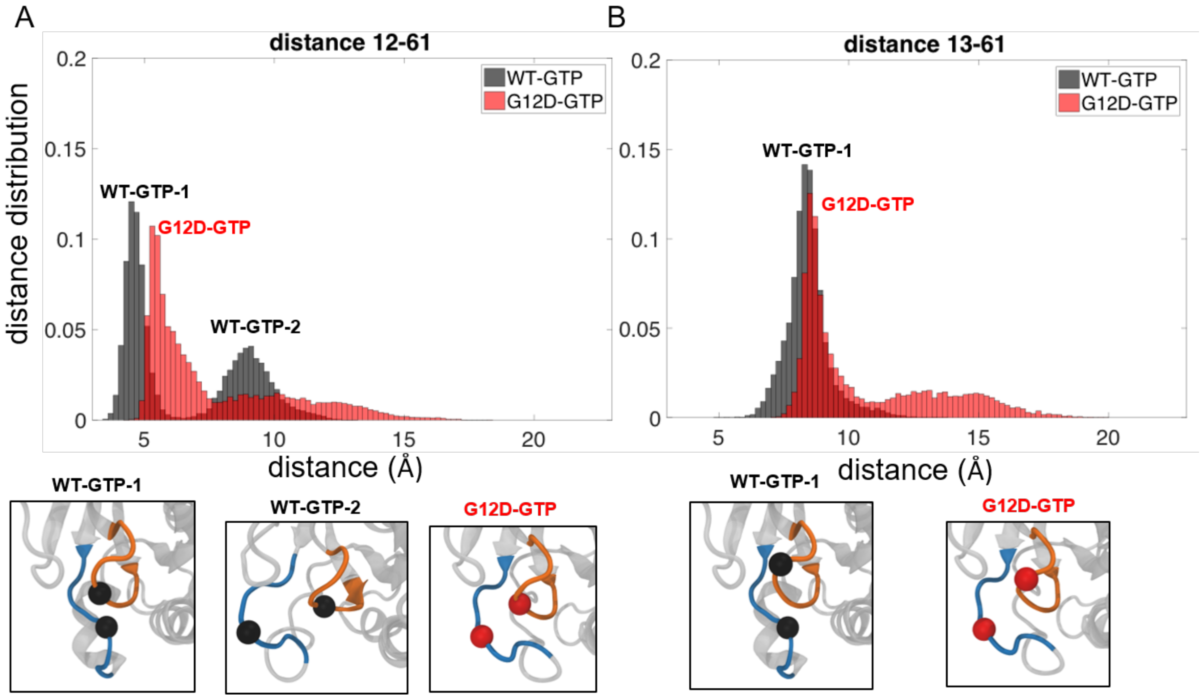

Figure S12 (Related to Figure 4B-C in the paper). Distance distributions between residue pairs in SII- P-loop region in K-Ras^{WT}-GTP (black) and K-Ras^{G12D}-GTP (red) and representative structures of SII (blue)-P loop (orange) (A) G12D-Q61, (C) G12D-Q61.

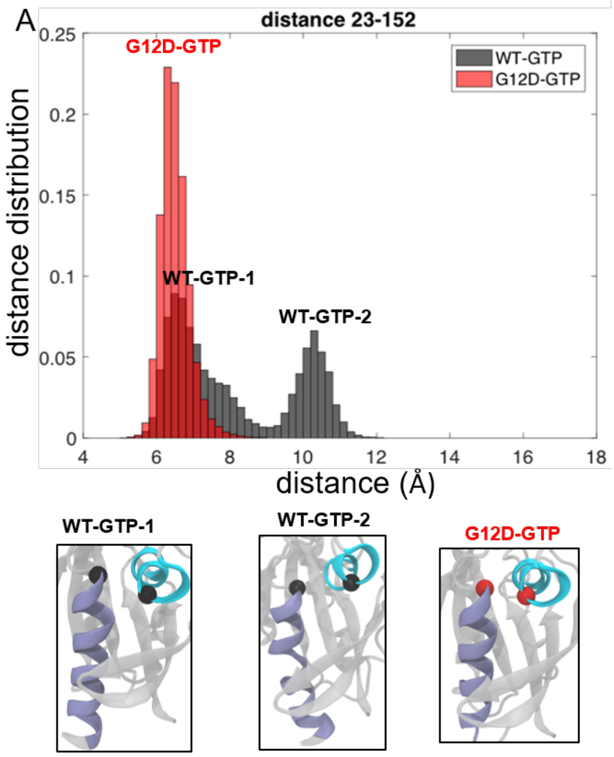

Figure S13 (Related to Figure 5B in the paper). Distance distributions of residue pairs which become closer after G12D mutation in K-Ras^{WT}-GTP (black) and K-Ras^{G12D}-GTP (red) and the representative structures of α2 (cyan), α5 (purple) (A) L23-V152.

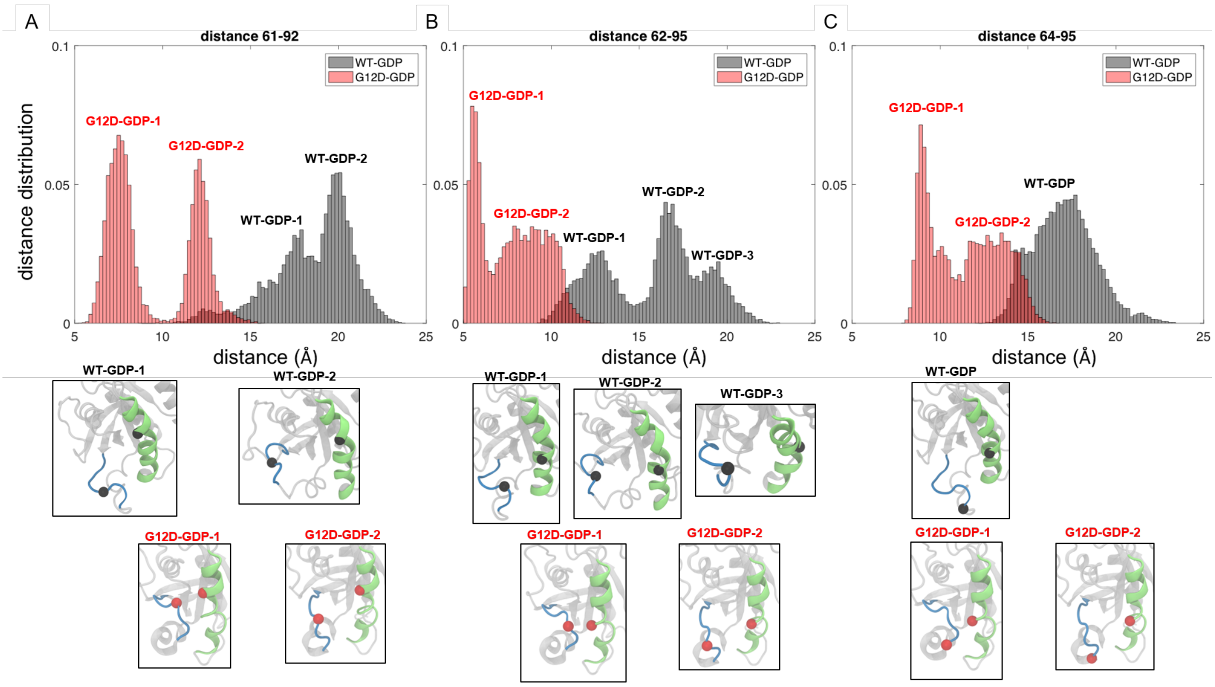

Figure S14 (Related to Figure 3D-F in the paper). Distance distributions between residue pairs in SII- α3 region in K-Ras^{WT}-GDP (grey) and K-Ras^{G12D}-GDP (pink) and the representative structures of SII (blue)-α3 (green) (A) Q61-D92E, (B) 62-H95, (C) Y64-H95.

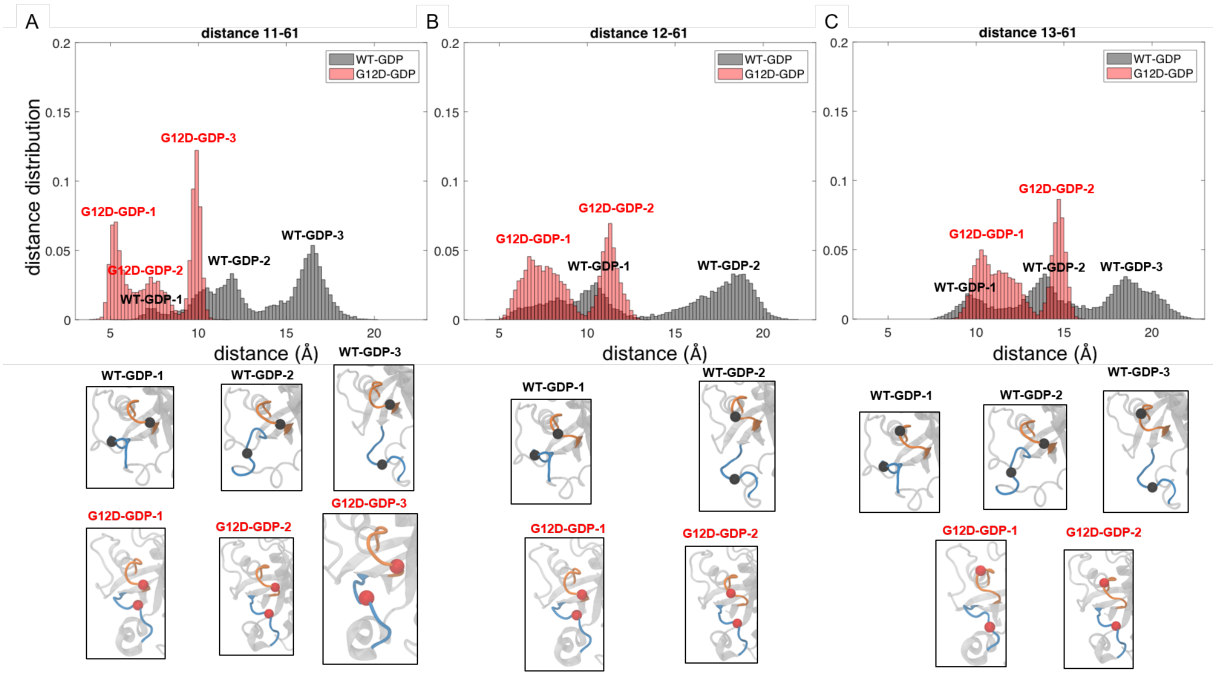

Figure S15 (Related to Figure 4D-F in the paper). Distance distributions between residue pairs in SII- P-loop region in K-Ras^{WT}-GDP (grey) and K-Ras^{G12D}-GDP (pink) and the representative structures of SII (blue)-P-loop (orange) (A) A11-Q61, (B) G12D-Q61, (C) G12D-Q61.

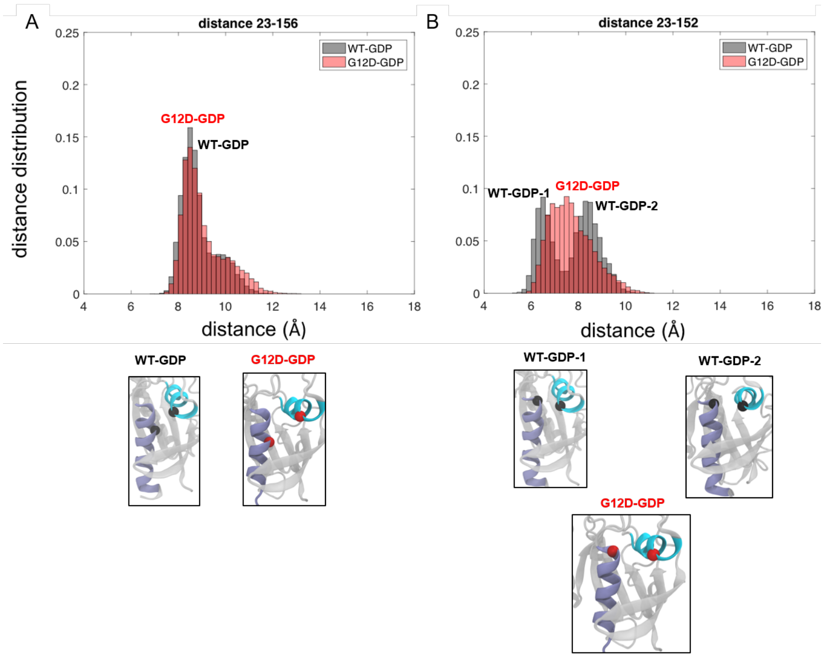

Figure S16 (Related to Figure 5C-D in the paper). Distance distributions of residue pairs which become closer after G12D mutation in K-Ras^{WT}-GDP (grey) and K-Ras^{G12D}-GDP (pink) and the representative structures of α2 (cyan), α5 (purple) (A) L23-V152, (B) L23-F156**.**

Supplementary Tables

Table S1. Sum of $\Delta\overline{R_{ij}}$ values

Residue Pair	Conformation in K-Ras^{G12D}	Residue Number	Sum of $\Delta\overline{R_{ij}}$ (Å)
(61, 62) -92	distant	61	5.60
		62	8.52
		92	13.00
64-95	distant	64	4.20
		95	7.70
(11, 12, 13)-61	distant	11	11.41
		12	7.50
		13	7.31
23-(152, 156)	closer	23	-3.92
		152	2.22
		156	-2.92
69-103	closer	69	3.66
		103	2.24

Table S2. The mean values of the normal distributions (μ) and the standard deviation values of the normal distributions (∑) of the residue pairs of GTP-bound K-Ras in Figures 3A-C, 4A-C and 5A-C.

Related Figure	Residue Pairs	WT-GTP		G12D-GTP	
		state-1	state-2	state-1	state-2
Figure 3A-C	61-92	μ = 8.48	μ = 16.1245		μ = 14.7125
		∑ = 1.00	∑ = 1.11352		∑ = 2.37368
	62-95	μ = 10.1162	μ = 16.7973		μ = 16.1576
		∑ = 1.14186	∑ = 1.89723		∑ = 1.86547
	64-95	μ = 9.68086	μ = 15.6928	μ = 11.8095	μ = 16.4179
		∑ = 1.4405	∑ = 0.941241	∑ = 0.864	∑ = 1.65205
Figure 4A-C	11-61	μ = 4.12218	μ = 12.8149	μ = 7.93337	μ = 13.064
		∑ = 0.349977	∑ = 0.935609	∑ = 0.883417	∑ = 1.91121
	12-61	μ = 4.67651	μ = 9.13938	μ = 7.93337	μ = 13.064
		∑ = 0.431423	∑ = 0.946378	∑ = 0.883417	∑ = 1.91121
	13-61	μ = 7.93337		μ = 8.07752	μ = 13.5322
		∑ = 0.883417		∑ = 1.04614	∑ = 1.7038
Figure 5A-C	23-156	μ = 8.71666	μ = 13.5315	μ = 8.73682	
		∑ = 0.511951	∑ = 0.933637	∑ = 0.391863	
	23-152	μ = 6.99846	μ = 10.2514	μ = 6.51076	
		∑ = 0.698898	∑ = 0.462543	∑ = 0.388253	
	69-103	μ = 6.99846		μ = 7.30826	
		∑ = 0.698898		∑ = 1.24021	

Table S3. The mean values of the normal distributions (μ) and the standard deviation values of the normal distributions (∑) of the residue pairs of GDP-bound K-Ras in Figures 3D-F, 4D-F and 5D-F.

Related Figure	Residue Pair	WT-GDP			G12D-GDP		
		state-1	state-2	state-3	state-1	state-2	state-3
Figure 3D-F	61-92	μ=16.33	μ=13.39		μ=7.51	μ=12.21	
		∑=1.72	∑=0.80		∑=0.68	∑=0.70	
	62-95	μ=12.44	μ=16.70	μ=19.42	μ=5.73	μ=8.88	
		∑=1.18	∑=0.68	∑=0.90	∑=0.35	∑=1.23	
	64-95	μ=17.04			μ=9.42	μ=13.10	
		∑=1.75			∑=0.68	∑=1.14	
Figure 4D-F	11-61	μ=7.29	μ=10.99	μ=16.14	μ=5.41	μ=7.49	μ=9.86
		∑=0.40	∑=1.13	∑=1.15	∑=0.42	∑=0.62	∑=0.28
	12-61	μ=9.17	μ=17.71		μ=7.40	μ=11.31	
		∑=1.71	∑=1.64		∑=1.01	∑=0.53	
	13-61	μ=9.81	μ=13.39	μ=18.33	μ=11.04	μ=14.68	
		∑=0.81	∑=0.80	∑=1.77	∑=1.01	∑=0.39	
Figure 5D-F	23-156	μ=8.44	μ=16.70		μ=8.58	μ=10.29	
		∑=0.32	∑=0.68		∑=0.39	∑=0.63	
	23-152	μ=6.55	μ=8.48		μ=7.70		
		∑=0.36	∑=0.54		∑=0.90		
	69-103	μ=7.27	μ=10.35	μ=13.72	μ=6.22	μ=9.63	
		∑=0.71	∑=0.86	∑=1.16	∑=0.98	∑=0.75	

Supplementary References

1 Vatansever, S., Gümüş, Z. H. & Erman, B. Intrinsic K-Ras dynamics: A novel molecular dynamics data analysis method shows causality between residue pair motions. *Sci Rep* **6**, 37012, doi:10.1038/srep37012 (2016).
